# Stroma derived COL6A3 is a potential prognosis marker of colorectal carcinoma revealed by quantitative proteomics

**DOI:** 10.18632/oncotarget.4966

**Published:** 2015-08-12

**Authors:** Jie Qiao, Cai-Yun Fang, Sun-Xia Chen, Xiao-Qing Wang, Shu-Jian Cui, Xiao-Hui Liu, Ying-Hua Jiang, Jie Wang, Yang Zhang, Peng-Yuan Yang, Feng Liu

**Affiliations:** ^1^ Department of Medical Systems Biology of School of Basic Medical Sciences, Shanghai, China; ^2^ Institutes of Biomedical Sciences Fudan University, Shanghai, China; ^3^ Department of Chemistry, Fudan University, Shanghai, China; ^4^ College of Bioscience and Biotechnology, Key Laboratory of Crop Genetics and Physiology of Jiangsu Province, Yangzhou University, Yangzhou, China

**Keywords:** colorectal cancer, tumor microenvironment, fibroblast, proteomics, COL6A3

## Abstract

Colorectal cancer (CRC) represents the third most common cancer in males and second in females worldwide. Here, we performed a quantitative 8-plex iTRAQ proteomics analysis of the secreted proteins from five colonic fibroblast cultures and three colon cancer epithelial cell lines. We identified 1114 proteins at 0% FDR, including 587 potential secreted proteins. We further recognized 116 fibroblast-enriched proteins which were significantly associated with cell movement, angiogenesis, proliferation and wound healing, and 44 epithelial cell-enriched proteins. By interrogation of Oncomine database, we found that 20 and 8 fibroblast-enriched proteins were up- and downregulated in CRC, respectively. Western blots confirmed the fibroblast-specific secretion of filamin C, COL6A3, COL4A1 and spondin-2. Upregulated mRNA and stroma expression of *COL6A3* in CRC, which were revealed by Oncomine analyses and tissue-microarray-immunohistochemistry, indicated poor prognosis. *COL6A3* expression was significantly associated with Dukes stage, T stage, stage, recurrence and smoking status. Circulating plasma COL6A3 in CRC patients was upregulated significantly comparing with healthy peoples. Receiver operating characteristic curve analysis revealed that COL6A3 has better predictive performance for CRC with an area under the curve of 0.885 and the best sensitivity/specificity of 92.9%/81.3%. Thus we demonstrated that COL6A3 was a potential diagnosis and prognosis marker of CRC.

## INTRODUCTION

Colorectal cancer (CRC) is the third and second most common cancer in males (746 thousand incidences) and females (614 thousand incidences), respectively, as reported by the GLOBOCAN [1]. The cancer mortality is 51% (694 thousand) of all CRC cases and 8.5% of all type of cancers. The incidence of CRC in China is 253 thousand in 2012, with an increased mortality rate of 55% [1]. Although the surgery and chemotherapy effectiveness have been improved greatly, the poor post-surgery outcome of CRC indicates an essentiality to diagnose CRC at the early stage. It also reflects a deficiency in prognosis evaluation to achieve an individual treatment of CRC patients. Therefore, there is an urgent need to identify reliable markers for early CRC diagnosis and prognosis evaluation after curable resection.

The tumor microenvironment influences tumorigenesis and metastasis of cancer cells. The microenvironment is composed of diverse type of cells, such as cancer cells, epithelial cells, immune cells and fibroblasts, as well as the soluble factors and extracellular matrix proteins released by these cells [2]. The fibroblasts represent the most abundant stromal cells surrounding cancer cells. The interactions between cancer cells and the microenvironment is complicated, as the stroma elements can provide a support or constraint niche for tumor growth or metastasis [3, 4]. In some cases, candidate colon cancer protein markers were subsequently identified to be derived from the cancer stroma instead of the cancer epithelia cells, emphasizing the importance of investigation of stromal expressions. Therefore, deciphering the specific components of cancer stroma might shed light on the tumor-microenvironment interaction and identify potential stroma-derived factor that could serve as diagnosis or prognosis biomarker of human cancer.

Proteomic technologies have been widely used in profiling the protein expression of cancer cell lines and cancerous tissues. The quantitative methods of the proteomic analysis have revolutionized from semi-quantitative label-free quantitation to precise quantitation based on stable isotope labeling of extracted protein samples, living cells and even living animals [5]. Two representative technologies were the stable isotope labeling with amino acids in cell culture (SILAC) and the isobaric tags for relative and absolute quantitation (iTRAQ). SILAC was suitable for dynamic labeling of living cells or animals with a limitation for labeling less than three samples, while iTRAQ was applied for extracted protein samples with a more robust labeling and detection capacity of eight samples in parallel. Recently, proteomic methods have been used to address the differential expression of the secreted proteins between colon cancer-associated fibroblast and normal fibroblast [6–8]. Karagiannis GS *et al* analyzed the secretome profiles in fibroblast-cancer cell coculture or monoculture systems with one normal fibroblast and two colon cancer cell lines [9]. Colon cancer cell secretome also represented a valuable and attractive source to find candidate biomarkers, as had being addressed by previous proteomic investigations [9–18]. Despite these efforts and advances, the differential expression between the stromal fibroblasts and cancer epithelial cells remains to be fully addressed quantitatively.

The aim of current study is to identify colonic fibroblast-enriched secreted proteins comparing with colon cancer epithelia cells by quantitative proteomic analysis and find potential stromal-derived diagnostic or prognostic protein markers for colon cancer. We used one normal colonic fibroblast cell line and four fibroblast cultures established from fresh operating samples of colorectal adenocarcinoma and neighboring normal mucosa [7]. For comparison purpose, we used three colon cancer cell lines of different genetic backgrounds. By comparing the fibroblast and colon cancer cell secretomes, we identified colonic fibroblast-enriched secreted proteins, from which we further revealed significantly up- or downregulated proteins by interrogating public gene microarray expression datasets. Thereafter, we performed additional experiments to analyze the diagnostic or prognostic significance of the most prominent candidate marker of colon cancer. Using such strategy, we identified 1114 proteins and classified 116 fibroblast-enriched proteins and 44 colon cancer cell-enriched proteins. Finally we revealed that collagen alpha-3(VI) chain (COL6A3) was a potential diagnostic and prognostic biomarker of colon cancer.

## RESULTS

### Differential expression analysis of stromal and epithelial cells of colorectal cancer using quantitative proteomic strategy

**Figure 1:**
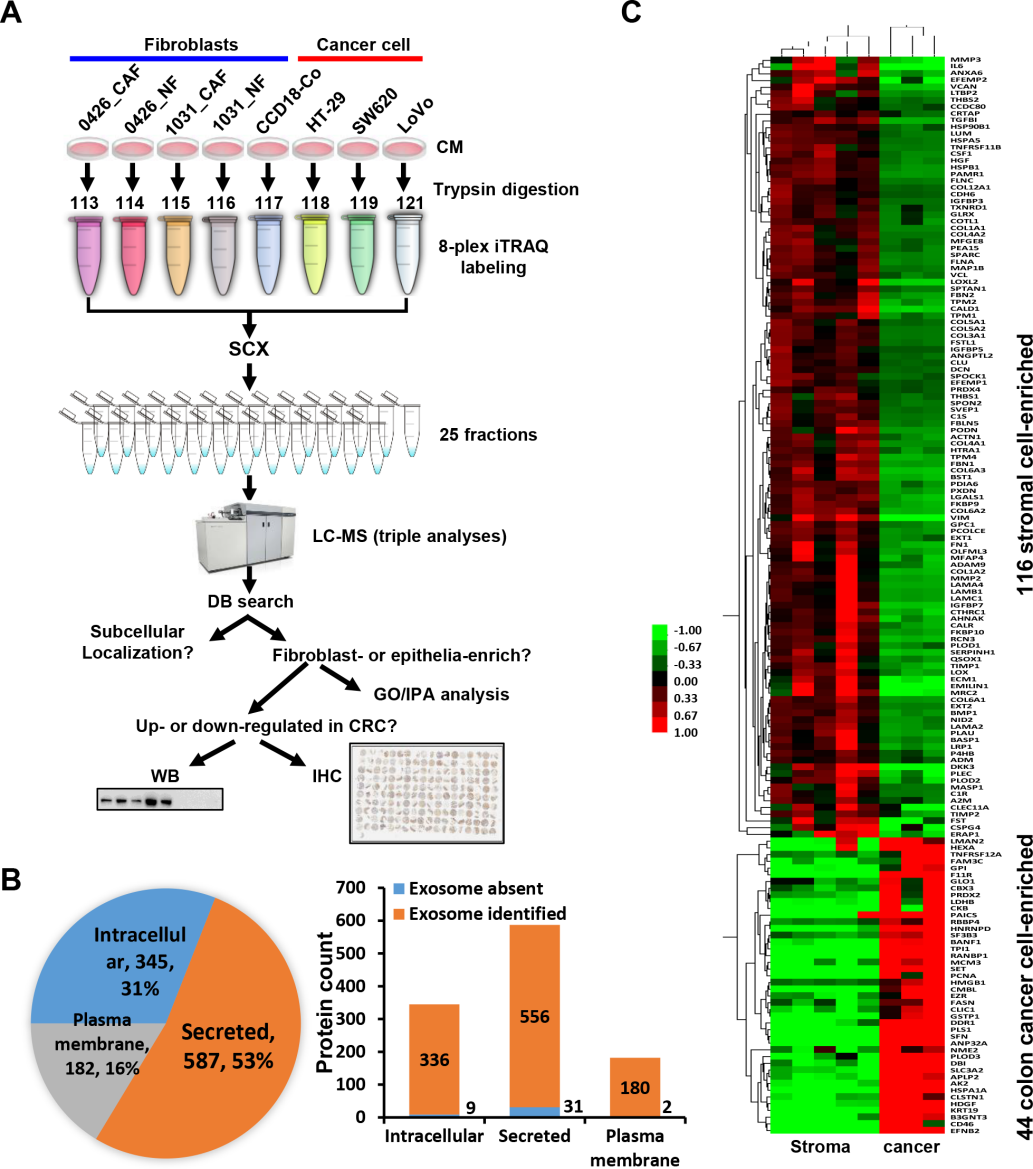
Quantitative proteomic analysis of the secretomes of colon cancer cells and the stromal cells **A.** The strategy of the 8-plex iTRAQ-LC-MS analysis of the secretomes of the fibroblast cell lines and colon cancer cell lines. NF, normal fibroblast; CAF, cancer-associated fibroblast; CM, conditioned medium. The sample was analyzed by LC-MS with triple loading. **B.** Left, the proportion of different subcellular localization of all identified proteins. Right, identified proteins presented in known exosome databases. **C.** The heat map of the firbroblast-enriched or epithelial cell-enriched proteins were generated using Cluster and TreeView.

We collected the conditioned media from the five fibroblast cell lines including 0426_NF, 0426_CAF, 1031_NF, 1031_CAF and CCD-18Co, and three colon cancer epithelial cell lines including SW620, HT-29 and LoVo. We labeled the secreted proteins of above cell lines with 8-plex iTRAQ reagents. We then performed a two-dimensional LC-MS analysis in triplicate and DB searching against UniProtKB/Swiss-Prot DB (Figure 1A). The raw DB searching results from the three experiments were combined according to the protein accession numbers. As determined by ProteomicS Performance Evaluation Pipeline (PSPEP) algorithm, the Unused ProScores that correspond to 1% FDR for the three experiments were 2, 1.62 and 2.01, respectively (Supplementary Table 1). The proteins having Unused ProScores exceeding above cutoffs and at least 2 unique peptides (95% probability) in at least one experiment were accepted. By this stringent filtering, all decoy matches were filtered, achieving a FDR of 0%. In total, we identified 1114 proteins, among which 825 proteins had quantitative values in all three experiments (Supplementary Table 2). The other 279 proteins were quantified in one or two of all three replicates. The remaining 10 protein had no quantitative iTRAQ values.

We analyzed the subcellular localization of the 1114 proteins using annotation or prediction results from UniProt, DAVID, exosome DBs, SignalP, Phobius and SecretomeP. There were 587 (53%), 182 (16%) and 345 (31%) proteins were recognized as secreted, plasma membrane-located and intracellular proteins, respectively (Figure 1B). We compared the proteins with exosome identifications in Vesiclepedia and ExoCarta databases [19, 20]. Strikingly, most proteins (>95%), including the intracellular proteins, were found in known exosome proteomes of human-origin samples analyzed by experimental methods including proteomics (Figure 1B).

### Characterization of the stroma- or cancer cell-enriched proteins

**Figure 2:**
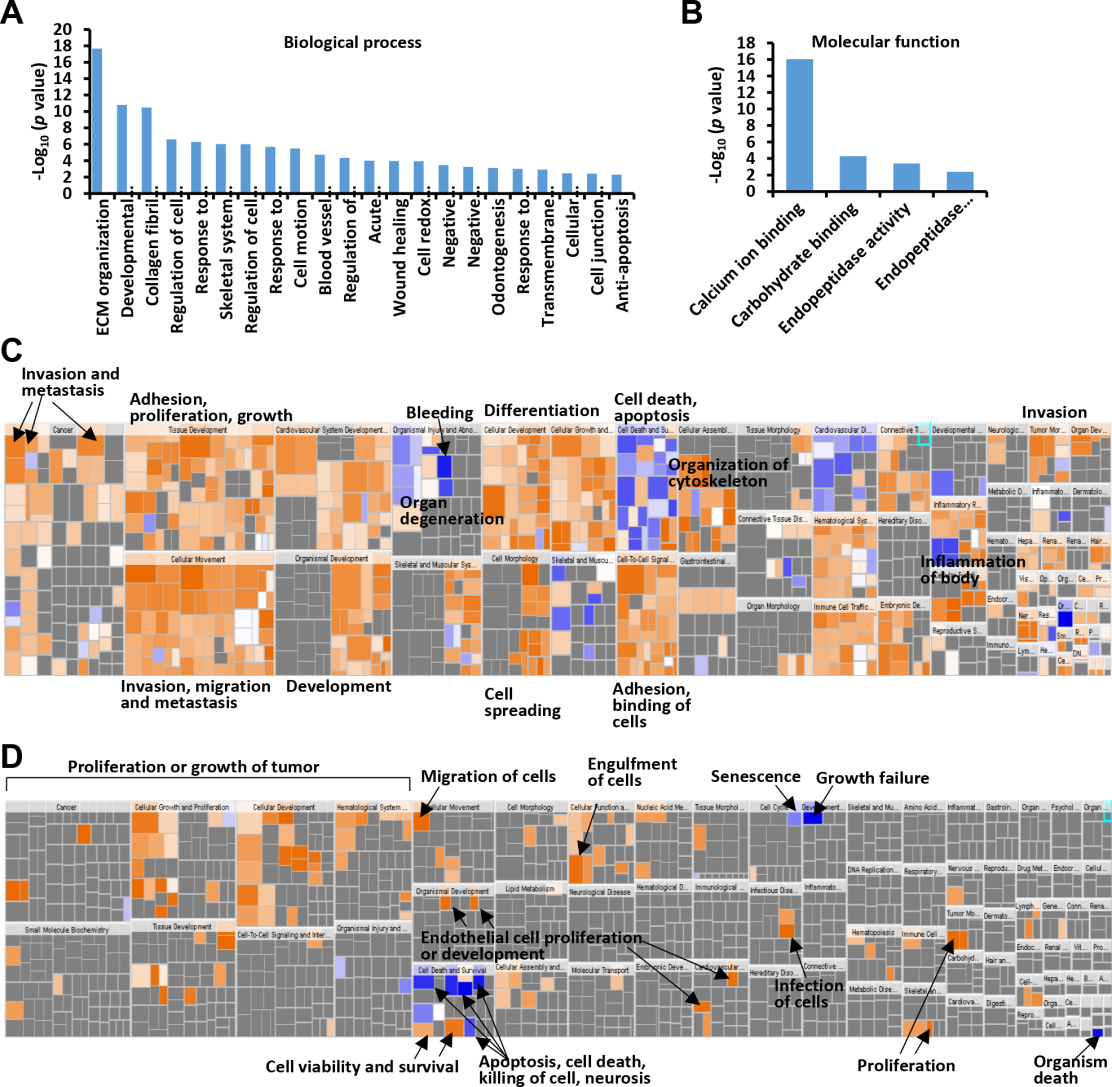
GO and IPA analysis of colonic fibroblast- or colon cancer cell-enriched proteins **A.** Biological process assignments of identified fibroblast-enriched proteins using DAVID (http://david.abcc.ncifcrf.gov/). **B.** Molecular function assignments of identified fibroblast-enriched proteins. **C.** Over-represented diseases and bio functions of colonic fibroblast-enriched proteins by IPA analysis. **D.** Over-represented diseases and bio functions of colon cancer cell-enriched proteins. The square size was determined by –log (*p*-value), while the color was defined by the z-score. The arrows illustrated the significantly enriched functional categories.

Of the 825 proteins with quantitative values in all three iTRAQ LC-MS datasets, 116 and 44 proteins were revealed to be fibroblast-enriched and cancer epithelial cancer cell-enriched, respectively (Figure 1C). We performed Gene Ontology (GO) function enrichment analysis of these proteins using the DAVID tool. For the 116 proteins, enriched biological process (BP) GO terms were grouped into 22 clusters (Supplementary Table 3). The top five BP clusters included extracellular matrix (ECM) organization, developmental process, collagen fibril organization, skeletal system development and response to wounding (Figure 2A). The enriched molecular function (MF) were 4 clusters, including calcium ion binding, carbohydrate binding, endopeptidase activity and endopeptidase inhibitor activity (Figure 4B). For the 44 epithelial cancer cell-enriched proteins, the enriched BP categories were anti-apoptosis, carbohydrate metabolic process, nitrogen compound metabolic process, anatomical structure development and organ development (Supplementary Table 3). Enriched MF clusters for the epithelial cancer cell-enriched proteins were protein binding (GO:0005515) and binding (GO:0005488) (Supplementary Table 3). These results indicated the functional divergence of the fibroblast cell and the epithelial cancer cell expressions.

We analyzed the over-represented diseases and bio functions of the fibroblast-enriched or epithelia-enriched proteins using Ingenuity Pathway Analysis (IPA) (Supplementary Table 4). The 116 fibroblast-enriched proteins were significantly associated with a broad array of physiological processes including vasculogenesis/angiogenesis, proliferation, cell movement, adhesion, differentiation and wound healing, highlighting the modulatory role of local fibroblasts in tumorigenesis and metastasis (Supplementary Table 4, Figure 2C). The epithelial colon cancer cell-enriched proteins were overrepresented for proliferation, binding, permeability, movement and cell survival (Supplementary Table 4, Figure 2D).

### Up or down-regulated fibroblast-enriched proteins in colon cancer

**Figure 3:**
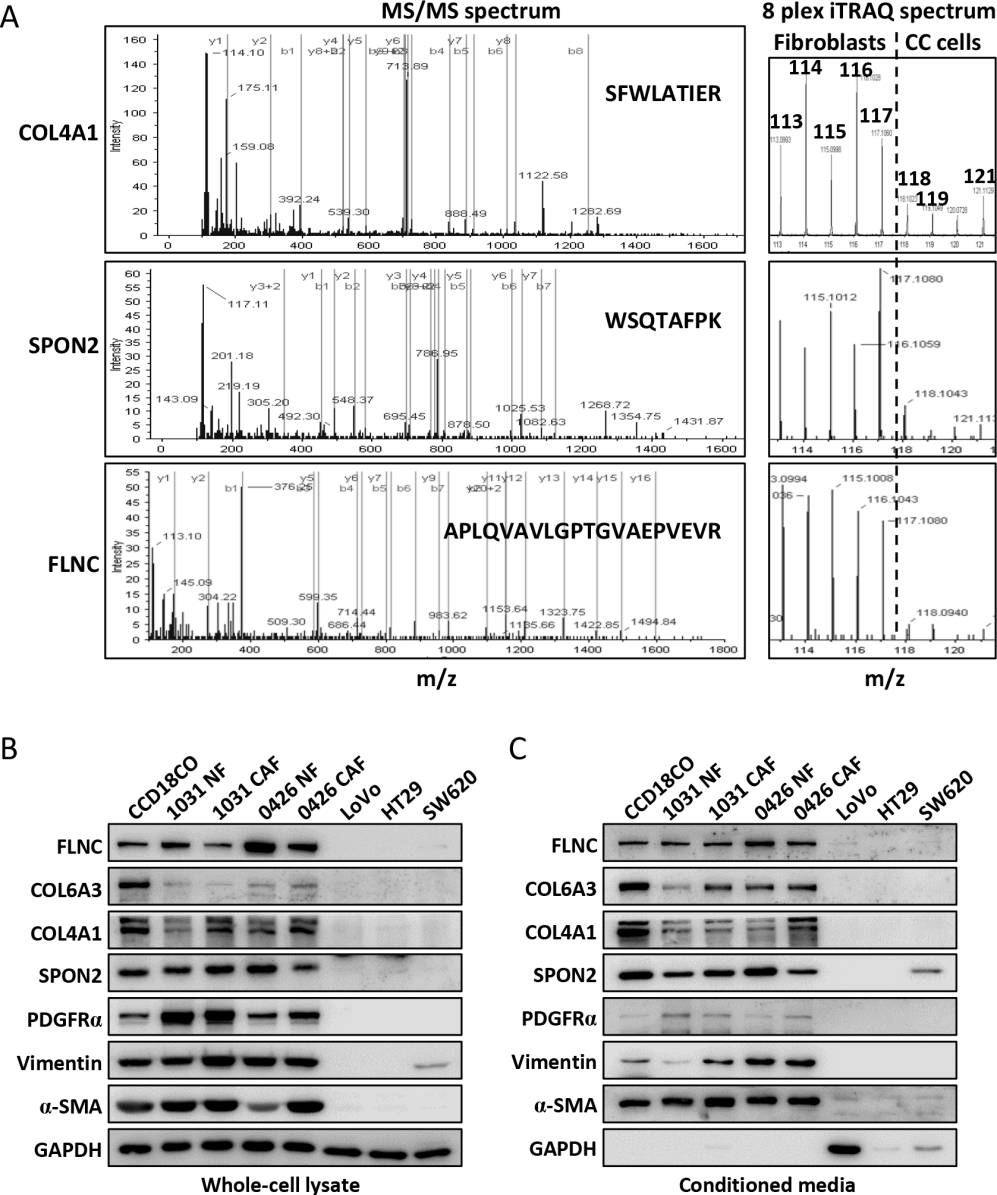
Representative tandem mass spectra of selected proteins and their expression verification in the fibroblasts and colon cancer cells **A.** The representative tandem mass spectra of COL4A1, SPON2 and FLNC were shown to the right side. The b and y series ion were indicated with green and red vertical lines, respectively. The peptide sequences were shown within the spectra. The iTRAQ ion spectra were extracted from the low mass region of the left spectra and shown to the right side. The dash line separated the ions labeling the fibroblasts from that of the colon cancer cell lines. **B.** The cytoplasmic expressions of the COL6A3, FLNC, COL4A1 and SPON2 in the fibroblasts and colon cancer cells were analyzed by Western blot. **C.** The expression levels of the selected proteins in the culture media of the fibroblasts and colon cancer cells. PDGFRα, vimentin and α-SMA were typical fibroblast markers.

We then ask if these fibroblast-enriched proteins were down- or upregulated in mRNA level in colon cancer by interrogating the Oncomine cancer microarray mRNA database. There were 20 corresponding genes were significantly upregulated in colon cancer as these genes were found to be upregulated in more than 15 microarray analyses but were downregulated in 0∼1 microarray analyses (Table 1 and Supplementary Table 2). Similarly, 8 corresponding genes were revealed to be considerably downregulated in colon cancer. Among the upregulated stromal cell-enriched proteins, some proteins have been reported to be potential markers in colon cancer prognosis or tumorigenicity, such as collagen XII (COL12A1) [9], thrombospondin 2 (THBS2) [21], collagen triple helix repeat-containing protein 1 (CTHRC1) [22] and COL5A2 [23]. We chose COL4A1, COL6A3, spondin-2 (SPON2) and FLNC for further analysis for their high abundance (peptide count), high “fold change” and less chances being identified in colon cancer cell lines (Supplementary Table 2). FLNC was a potential secretory protein as it was frequently detected in exosome analyses (32 experiments) (Supplementary Table 2), although it was predicted to be an intracellular protein. The representative tandem spectra and iTRAQ tag spectra of COL4A1, SPON2 and FLNC were shown in Figure 3A. The Western blot assay indicated that FLNC, COL6A3, COL4A1 and SPON2 were abundantly expressed in the colonic fibroblasts instead of the colon cancer epithelial cells (Figure 3B). Their expression patterns resembled the three typical fibroblast markers, PDGFRα, vimentin and α-SMA (Figure 3B). In the conditioned media, FLNC, COL6A3, COL4A1 and SPON2 were mainly fibroblast-enriched (Figure 3C). These results indicated a successful performance of the quantitative proteomic analysis. Interestingly, GAPDH was absent in the CMs of the fibroblasts but present in the CMs of LoVo and SW620. A previous analysis indicated that intracellular GAPDH could be secreted in the CM of some mammalian cell lines including cancer cell lines and might induce cell morphological change or inhibit cell spreading [24].

**Table 1:** Stromal cell-enriched proteins that are up- or downregulated in colon cancer

Accession	Name	Unused Score^a^	%Cov (95)^b^	Peptides (95%)^c^	Average fold change^d^	In colorectal cancer^e^		
						**Up**	**Down**	**Total**
P02462	COL4A1 (Collagen alpha-1(IV) chain)	17.7	10.5	13	10.1	21	–	26
P05997	COL5A2 (Collagen alpha-2(V) chain)	50.2	29.9	37	30.5	19	–	23
P07942	LAMB1 (Laminin subunit beta-1)	81.8	28.3	50	22.2	16	–	27
P12111	COL6A3 (Collagen alpha-3(VI) chain)	188.9	40.0	116	26.9	19	–	26
P35442	THBS2 (Thrombospondin-2)	4.4	5.5	6	8.7	19	–	26
Q02809	PLOD1 (Procollagen-lysine,2-oxoglutarate 5-dioxygenase 1)	37.1	37.5	23	3.3	19	1	24
Q15084	PDIA6 (Protein disulfide-isomerase A6)	16.1	26.8	9	4.4	20	–	23
Q16610	ECM1 (Extracellular matrix protein 1)	24.6	30.7	17	10.7	17	–	25
Q96AY3	FKBP10 (Peptidyl-prolyl cis-trans isomerase FKBP10)	24.3	31.3	14	12.1	15	–	24
Q96CG8	CTHRC1 (Collagen triple helix repeat-containing protein 1)	4.5	11.5	3	6.3	20	1	23
Q99715	COL12A1 (Collagen alpha-1(XII) chain)	142.8	35.1	85	17.0	22	1	23
Q9BUD6	SPON2 (Spondin-2)	26.5	58.3	17	9.8	20	–	24
Q9Y240	CLEC11A (C-type lectin domain family 11 member A)	14.6	26.0	7	4.3	15	–	23
P00749	PLAU (Urokinase-type plasminogen activator)	12.8	25.1	10	13.9	23	–	25
P01033	TIMP1 (Metalloproteinase inhibitor 1)	55.9	72.0	80	6.0	24	–	25
P08254	MMP3 (Stromelysin-1)	28.7	29.1	16	8.2	24	–	24
P67936	TPM4 (Tropomyosin alpha-4 chain)	12.2	31.0	12	11.1	23	–	27
Q13162	PRDX4 (Peroxiredoxin-4)	4.0	13.3	4	2.2	24	–	26
Q15121	PEA15 (Astrocytic phosphoprotein PEA-15)	10.0	62.3	5	4.5	23	1	27
Q15582	TGFBI (Transforming growth factor-beta-induced protein ig-h3)	51.1	51.4	52	13.0	24	–	26
O00469	PLOD2 (Procollagen-lysine,2-oxoglutarate 5-dioxygenase 2)	16.3	20.4	13	6.2	–	15	25
P01023	A2M (Alpha-2-macroglobulin)	35.5	21.4	25	3.1	1	26	27
P55083	MFAP4 (Microfibril-associated glycoprotein 4)FF	6.0	13.7	3	5.1	–	18	25
Q10588	BST1 (ADP-ribosyl cyclase/cyclic ADP-ribose hydrolase 2)	3.9	7.5	2	8.7	–	20	26
Q14019	COTL1 (Coactosin-like protein)	9.1	26.8	6	4.0	1	15	24
Q14315	FLNC (Filamin-C)	79.4	24.5	49	18.1	1	15	24
Q16394	EXT1 (Exostosin-1)	14.6	14.3	10	4.7	–	23	25
Q9UBX5	FBLN5 (Fibulin-5)	13.0	18.8	7	8.9	1	16	24

a Unused (ProtScore) is calculated by ProteinPilot software from the peptide confidence for peptides from spectra that are not already completely “used” by higher scoring winning proteins. This is the maximal value in all three LC-MS runs.

b % Cov (95) indicates the percentage of matching amino acids from identified peptides having confidence > = 95% divided by the total number of amino acids in the sequence. This is the maximal value in all three LC-MS runs.

c Peptides (95%) is the number of distinct peptides having at least 95% confidence calculated by ProteinPilot. This is the maximal value in all three LC-MS runs.

d The average fold change indicates the comparison between the fibroblasts and the epithelial cancer cells.

e The mRNA expression of the identified protein is analyzed using Oncomine database (https://www.oncomine.org). The numbers indicate the analysis numbers that exceeding the threshold *p* value 0.05. The fold change and gene rank are set as “all” and the data type is restricted to mRNA.

**Figure 4:**
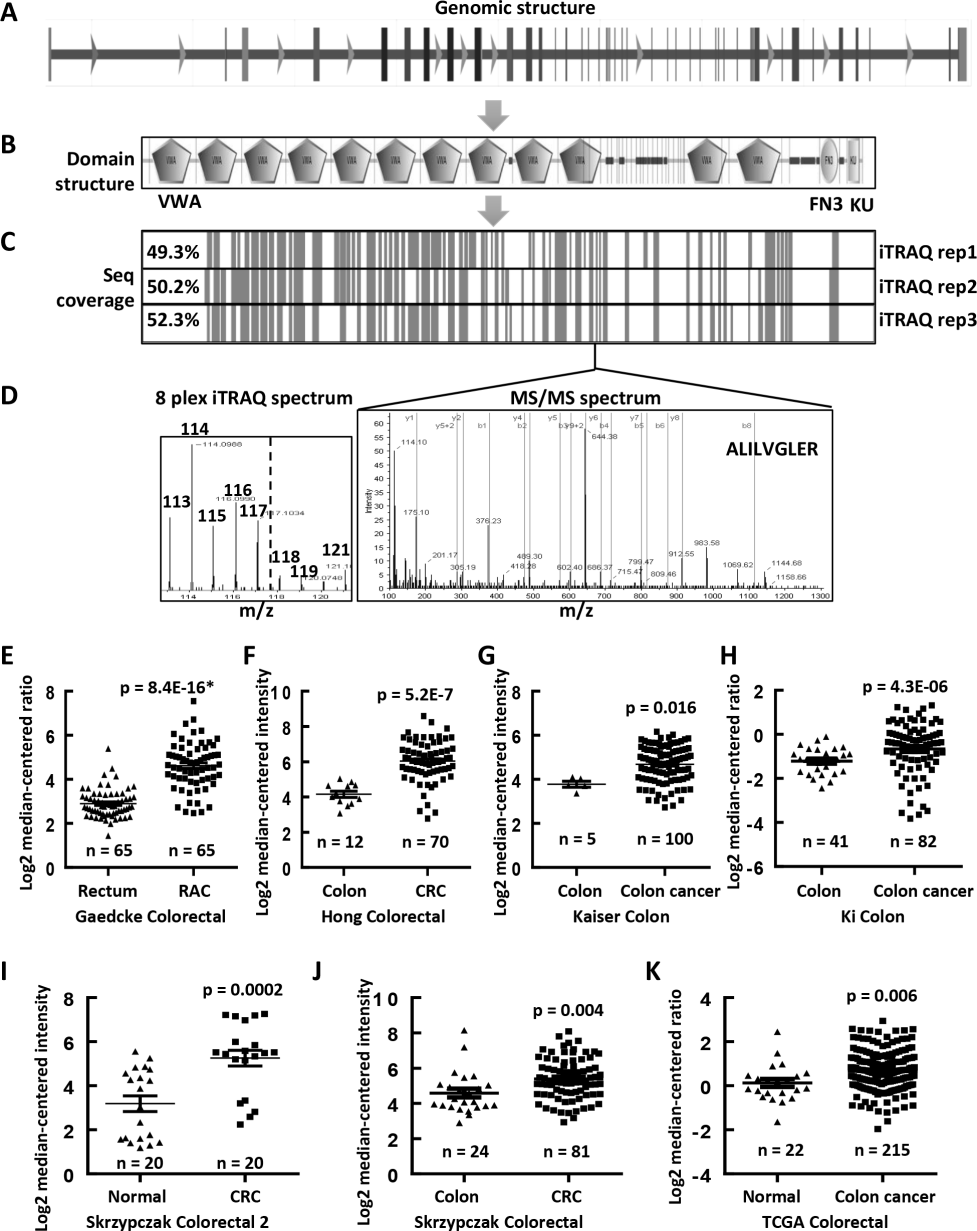
Upregulated mRNA expression levels of COL6A3 in colon cancers revealed by MS and Oncomine analyses **A.** The genomic structure of *COL6A3* gene contained 44 exons. **B.** The protein structure of COL6A3, as analyzed by SMART (http://smart.embl.de), contained 12 von Willebrand factor type A domain (VMA), one fibronectin type 3 domain (FN3) and one KU domain. **C.** The sequence coverage of COL6A3 by MS analysis. The vertical lines indicated the peptides identified in all replicated iTRAQ LC-MS. **D.** A represented MSMS spectra of a peptide of COL6A3, which was identified in all LC-MS experiments. The iTRAQ spectrum was shown to the left, with a dash line separating the tags labeling the fibroblasts and the colon cancer cell lines. *COL6A3* mRNA expression was found to be upregulated in colon cancers comparing with normal colonic tissues revealed by data-mining of gene expression microarray datasets deposited in Oncomine DB (https://www.oncomine.org/) including **E.** Gaedcke Colorectal [27], **F.** Hong Colorectal [28], **G.** Kaiser Colon [29], **H.** Ki Colon [30], **I.** Skrzypczak Colorectal 2 [31], **J.** Skrzypczak Colorectal [31] and **K.** TCGA Colorectal. Skrzypczak Colorectal 2 contains a subset of selected samples from Skrzypczak Colorectal further treated with micro-dissection. The case number of each category was listed under the dot. The significances are calculated using two-ways of Student's *t* tests for comparing between normal and tumor samples. The *p* values marked with an asterisk were calculated using paired Student's *t* test. A *p* value < 0.05 was considered as statistically significant. RAC, rectal adenocarcinoma; CRC, colorectal carcinoma.

COL6A3 has the most peptides identified, which was selected for further analysis (Table 1). Its gene expression was shown to be associated with the progression of breast cancer and was elevated in colon cancer [25, 26]. COL6A3 gene locates 2q37 in the human genome and contains 44 exons (Figure 4A). The COL6A3 protein has 3177 amino acids and contains 12 Von Willebrand factor type A (vWA) domains, one fibronectin type 3 domain and one BPTI/Kunitz family of serine protease inhibitors (KU) domain (Figure 4B). The sequence coverage of this protein ranged from 49.3% to 52.3% in all three LC-MS (Figure 4C). A representative tandem spectrum of COL6A3 was shown in Figure 4D.

### COL6A3 mRNA and protein were significantly upregulated in colon cancers

We performed a data mining analysis of the mRNA expression of *COL6A3* in colon cancers using the Oncomine gene expression array datasets. As shown in Figure 4E, *COL6A3* mRNA was found to be significantly upregulated in rectal adenocarcinoma tissues comparing with normal rectum tissues (*p* = 8.4E-16), as revealed by analyzing Gaedcke Colorectal dataset [27]. Similarly, we found that *COL6A3* gene expression was increased in colorectal cancer tissues comparing with normal tissues by analyzing Hong Colorectal (*p* = 5.2E-7) [28], Kaiser Colon (*p* = 0.016) [29], Ki Colon (*p* = 4.3E-6) [30], Skrzypczak Colorectal 2 (*p* = 0.0002) [31], Skrzypczak Colorectal (*p* = 0.004) [31] and TCGA Colorectal (*p* = 0.006) (Figure 4F–4K). All enrolled cases including 510 colon cancers and 189 normal specimen.

**Figure 5:**
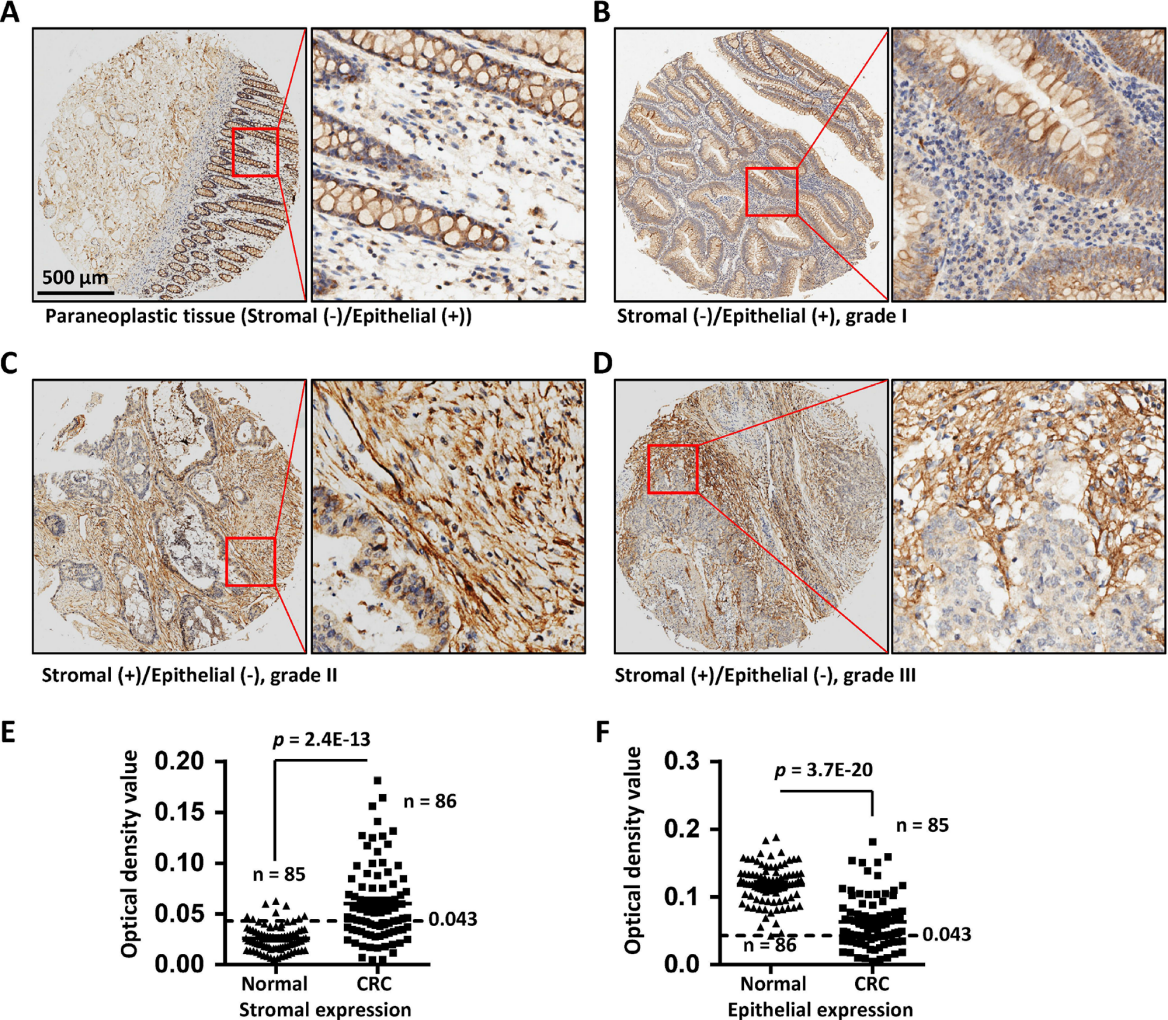
COL6A3 protein was upregulated in colorectal cancer stroma revealed by IHC analysis of a tissue microarray **A.** The paraneoplastic tissue of a colon cancer patient shows negative staining in the stroma cells and positive staining in the epithelial cells for COL6A3. The red rectangle illustrates the position of a snapshot shown in the right side. **B.** A grade I colorectal adenocarcinoma sample showed epithelial-positive but stromal-negative staining for COL6A3. **C.** A grade II colorectal adenocarcinoma specimen displayed stromal-positive but epithelia-negative staining of COL6A3 protein. **D.** A grade III colorectal adenocarcinoma specimen displayed stromal-positive but epithelia-negative staining of COL6A3. **E.** Stromal expression of COL6A3 in the normal and cancerous tissues was determined by analyzing the optical density of antibody stainings using Image Pro Plus. The epithelial regions were excluded during analyzing. N indicated the number of case enrolled for optical density scanning. Samples without visible stromal or epithelial cells were excluded from analysis. The dash line indicates the cutoff to differentiate the negative and positive staining. **F.** Epithelial expression of COL6A3 in the normal and cancerous tissues was analyzed by optical density scanning. The Stromal area was blocked before analyzing. The significances are calculated using two-ways of Student's *t* tests for comparing between normal and tumor samples. A *p* value < 0.05 was considered as statistically significant.

To further evaluate COL6A3 expression in colon cancer, we performed an immunohistochemistry (IHC) analysis of COL6A3 expression using a commercial tissue microarray (TMA) containing an independent cohort of CRC (90 cases) samples (Supplementary Table 5). We measured the integrated optical density (IOD) values of one or more area of each scanned TMA sample images. The epithelial or stromal region was analyzed separately by blocking the unanalyzed area using Image-Pro Plus (Supplementary Figure 1). In the paraneoplastic mucosa tissues, the epithelial adsorptive cells were stained positive for COL6A3, but the stromal cells were stained negative for COL6A3 (Figure 5A). In cancerous tissues, 48 specimens were stained positive for COL6A3 in the stromal area, while 31 specimens were stained negative for COL6A3 in the stromal regions. Furthermore, 30 specimens were COL6A3-negative and 56 specimens were COL6A3-positive in the epithelial regions. A representative specimen of grade I colorectal adenocarcinoma was presented in Figure 5B, showing that epithelial was positive while stroma was negative for COL6A3. Two cases of colorectal adenocarcinoma with COL6A3-positive stroma were displayed in Figure 5C and 5D. We revealed that stromal expression of COL6A3 was upregulated significantly in the CRC tissues comparing with the normal tissues (*p* = 2.4E-13) (Figure 5E), whereas the epithelial expression of COL6A3 was downregulated in the CRC tissues (*p* = 3.7E-20) (Figure 5F). This finding was in line with the quantitative LC-MS and Western blot results that COL6A3 was a cancerous-stromal cell-enriched protein.

### High COL6A3 expression is associated with prognosis of colon cancer patients

**Figure 6:**
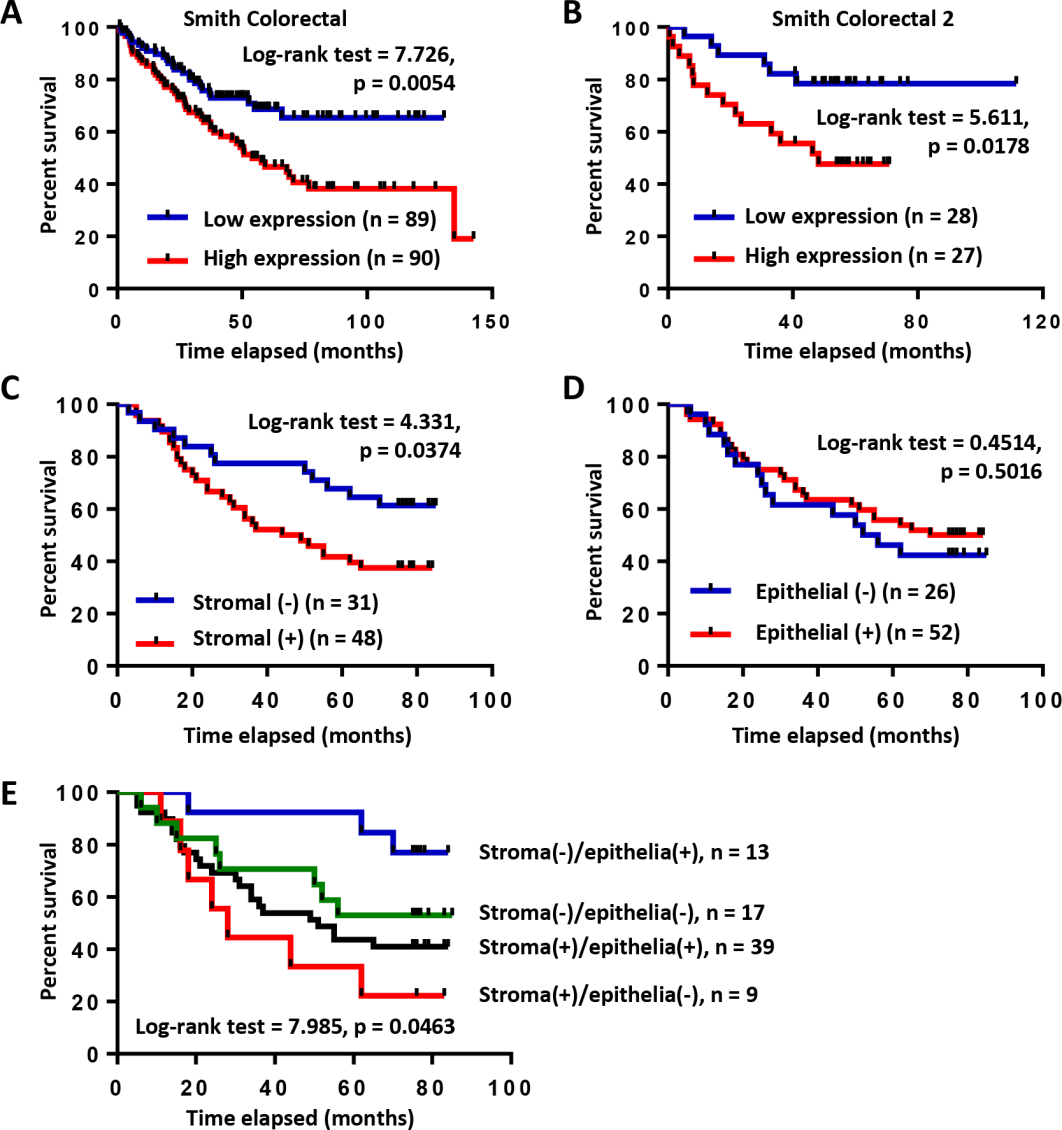
High expression of *COL6A3* mRNA and protein is associated with poor prognosis of colon cancer patients **A.** Kaplan-Meier survival curve analysis of colon cancer patients with *COL6A3* mRNA higher-than-median or lower-than-median using Smith Colorectal dataset from Oncomine database [32]. **B.** Kaplan-Meier survival curve analysis of colon cancer patients with *COL6A3* mRNA higher-than-median or lower-than-median using Smith Colorectal 2 dataset from Oncomine database [32]. **C.** Kaplan-Meier survival curve analysis of colon cancer patients with stromal-negative or stromal-positive expression of COL6A3 protein revealed by IHC analysis of a tissue microarray. The scoring was based on optical density calculation and manual scoring. **D.** Kaplan-Meier survival curve analysis of colon cancer patients with epithelia-negative or epithelia-positive expression of COL6A3 protein. **E.** Kaplan-Meier survival curve analysis of colon cancer patients of four categories based on COL6A3 expression in the stroma and cancer epithelia. The *p* value was calculated using the Log-rank (Mantel-Cox) test.

To address the prognosis significance of COL6A3 in CRC, we performed Kaplan-Meier survival curve analysis of colon cancer patients with different *COL6A3* mRNA level. Interestingly, high level of *COL6A3* was found to be associated with poor survival of colorectal cancer patients, as revealed by the Smith Colorectal dataset [32] (Log-rank test = 7.726, *p* = 0.0054) (Figure 6A) and the Smith Colorectal 2 dataset [32] (Log-rank test = 5.611, *p* = 0.0178) (Figure 6B), suggesting that upregulated *COL6A3* gene expression predicted poor prognosis of colon cancer patients.

Furthermore, we analyzed the relationship of COL6A3 protein expression with the outcome of CRC patients based on the TMA and IHC analysis. First, we analyzed the correlation of stromal COL6A3 expression with the survival of colon cancer patients. Interestingly, stroma-positive COL6A3 was significantly associated with poor prognosis of colon cancer patients (Log-rank test = 4.331, *p* = 0.0374) (Figure 6C). However, epithelial expression of COL6A3 protein had no significant association with the prognosis of colon cancer patients (Log-rank test = 0.4514, *p* = 0.5016) (Figure 6D). Stroma (+)/epithelia (−) expression of COL6A3 significantly associated with worse survival comparing with other three kinds of expression combinations (Log-rank test = 7.985, *p* = 0.0463), further highlighting the importance of stroma-specific COL6A3 expression as a prognosis indicator (Figure 6E). These results suggested that COL6A3 was a potential stroma-specific prognosis marker of colon cancer patients.

### Association of COL6A3 expression with clinic parameters of colon cancer patients

We analyzed the correlation between the mRNA expression of *COL6A3* and the clinicopathological parameters of colon cancer patients by re-analyzing selected Oncomine datasets containing more than 100 cases. The CRC cases were divided into two categories, higher-than-median and lower-than-median. We found that the high level of *COL6A3* mRNA was significantly corrected with the progression of Dukes stage (*p* = 0.001), stage (*p* = 0.002) and T stage (*p* = 0.025) in Bittner Colon dataset (Supplementary Table 6). *COL6A3* mRNA increased obviously in smoking patients (*p* = 0.02). The *COL6A3* expression was also associated with recurrence status of colon cancer patients (*p* = 0.006), as revealed by Smith Colorectal dataset [32].

We also analyzed the association of the protein expression of COL6A3 with the clinicopathological parameters of CRC patients. Positive COL6A3 in cancer stromal region was significantly associated with shorter survival time (Fisher's exact test, *p* = 0.0326) (Supplementary Table 7). Association of COL6A3 with other clinicopathological factors did not exceed the significance level, due possibly to the limited case number as compared with the Oncomine datasets analyzed.

### Circulating COL6A3 is a potential plasma marker of colorectal cancer

**Figure 7:**
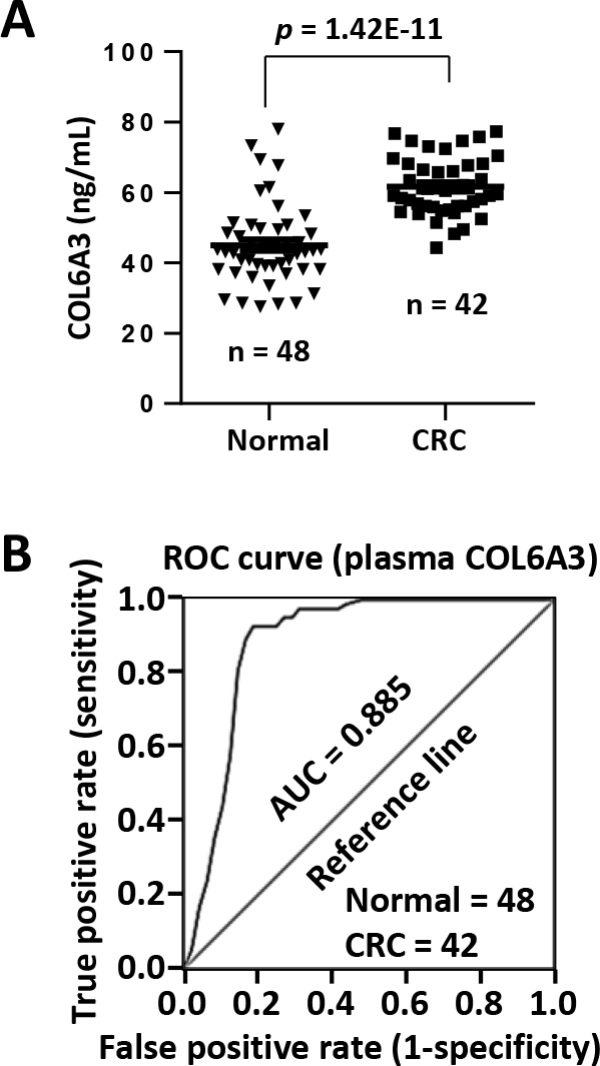
Circulating COL6A3 protein is a potential plasma marker of CRC **A.** Plasma concentration of COL6A3 in 42 CRC patients and 48 healthy donors was analyzed using ELISA. **B.** ROC curve analysis of plasma COL6A3 in CRC patients and healthy peoples. AUC, area under curve.

COL6A3 is a secreted protein. We then analyzed the plasma concentration of COL6A3 in CRC patients. The plasma COL6A3 in most healthy peoples remained at a constant and low level of 45.0 ng/mL (*n* = 48) (Figure 7A). However, COL6A3 was upregulated significantly in CRC patients (*n* = 42) (61.6 ng/mL) (*p* = 1.42E-11). We performed a receiver operating characteristic (ROC) curve analysis and found that COL6A3 displayed an area under the curve (AUC) value of 0.885, indicating that COL6A3 could be used to distinguish CRC patients from the healthy controls (Figure 7B). COL6A3 of concentration 51.2 ng/mL achieved the best sensitivity of 92.9% and the best specificity of 81.3%.

## DISCUSSION

Previously, we have analyzed the expression of colonic fibroblasts [7, 33]. However, it was quite limited as only one paired fibroblasts were analyzed and the information could be biased and restricted. In this study, we performed a quantitative 8-plex iTRAQ-LC-MS analysis of the secreted proteins from 5 colonic fibroblast cell lines and 3 colon cancer cell lines. The primary cell lines might lost their naive features after multiple passages, so it would not suitable for the analysis of the differential expression between normal and cancer-associated fibroblasts. Instead, we attempted to address the expression difference between the fibroblasts and the epithelial cancer cells, in spite of the origins of the fibroblasts. Such analysis could also shed light on the colonic microenvironment of colon cancer. The fibroblasts preserved their fibroblast characteristics as revealed by the Western blot analysis of typical fibroblast markers. Next, we could use gene array dataset as an orthogonal screen tool to identify “cancer-associated” proteins which were up- or down-regulated in cancer.

We identified and quantified 1114 proteins at 0% FDR including 53% secreted proteins and 16% plasma membrane protein, suggesting an effective isolation and identification of the secretomes. Although other 31% proteins were predicted to be intracellular proteins, most identified proteins were frequently detected in exosome identification analyses, suggesting some of the intracellular proteins might be released via unknown pathways. The current annotation and prediction tools might fail to identify atypical but genuine secretory proteins.

We recognized 116 stromal cell-enriched proteins and 44 epithelial cell-enriched proteins. Interestingly, the stromal cell-enriched proteins were found to be over-represented in functional categories of cell movement, migration, invasion and spreading, such as dickkopf (DKK3) [34], MMP3 [35], follistatin-related protein 1 (FSTL1) [7] and filamin A (FLNA) [36]. Whereas the cancer epithelial cell-enriched proteins were characterized of proliferation feature, such as hepatoma-derived growth factor (HDGF) [37], bone morphogenetic protein 7 (BMP7) and tumor protein D52 [38]. These observations highlighted the significance of our current analysis, as such comparison would shed light on the molecular microenvironment of solid malignancies.

We revealed that 28 coding genes among the 116 proteins were dysregulated in colon cancer by re-analyzing the Oncomine gene microarray datasets of colon cancers. We verified the iTRAQ-LC-MS results using Western blot to analyze 1 downregulated (in colon cancer) and 3 upregulated (in colon cancer) stromal-enriched proteins. The Western blot results of these proteins were consistent with the iTRAQ-LC-MS quantitation. Our results suggested that the public available gene microarray datasets of human cancers could provide an orthogonal screening tools to narrow down the candidates by proteomic analysis.

In current study, we chose COL6A3, an ECM protein, for further analysis. COL6A3 or its cleavage fragment (endotrophin) was found to be associated with cisplatin resistance [39, 40]. Stromal COL6A3 could promote tumor growth by modulating Hippo and Wnt signaling [41]. COL6A3 protein and mRNA were significantly upregulated in pancreatic ductal adenocarcinoma (PDA) and the diagnostic and prognostic value of circulating COL6A3 in PDA had been addressed [42, 43]. These findings indicated that COL6A3 might play a role in human cancer. In colon cancer, the upregulation of *COL6A3* gene and its alternatively splicing has been observed [25, 44]. *COL6A3* was associated with metastasis potential of single cell-derived progenies of colon cancer cell line SW480 [45]. A mutated *COL6A3* gene could predict better survival of colon cancer patient [46]. Despite all these observations, the diagnostic and prognostic value of COL6A3 in colon cancer remains elucidative. Here, we demonstrated that *COL6A3* mRNA was dramatically upregulated in CRC tissues comparing with normal tissues by re-analyzing Oncomine gene microarray datasets. By TMA-IHC analysis, we further revealed that COL6A3 protein was significantly upregulated in the stromal cells of CRC tissues comparing with the normal counterparts. Interestingly, the upregulation of COL6A3 in cancer stroma predicted poor outcome of CRC patients, as revealed by the Kaplan-Meier survival analysis. Similarly, overexpression of *COL6A3* mRNA was also associated with poor prognosis of CRC patients. These collective evidences suggested that COL6A3 was a stroma-specific prognosis marker of CRC. We revealed that *COL6A3* mRNA expression was significantly associated with Dukes stage, T stage, overall stage, recurrence status and smoking status of CRC patients, suggesting *COL6A3* might be served as diagnosis maker of CRC patients. ELISA assay indicated the circulating COL6A3 was significantly upregulated in CRC patient plasma and could be used as a diagnostic marker for having a prediction AUC value of 0.885, a sensitivity of 92.9% and a specificity of 81.3%.

In summary, we performed a quantitative iTRAQ-LC-MS analysis of the secretomes of colonic fibroblasts and colon cancer cell lines and identified CRC stroma-enriched and epithelia-enriched secretory proteins. Our study provides a novel and comprehensive insight into the molecular microenvironment of CRC. We demonstrated that COL6A3 would be a potential diagnosis and prognosis protein marker of CRC. These findings will be useful to develop potential therapeutic drug target or clinic biomarkers.

## MATERIALS AND METHODS

### Cell lines and cell culture

The primary fibroblast cultures used in current study, 1031_NF, 1031_CAF, 0426_NF and 0426_CAF, were established previously [7]. The fibroblasts were isolated from fresh cancerous and adjacent normal mucosa tissues (at least 5 cm away from the loci of cancerous tissue) within half an hour after tissue collection from two patients with colon ulcerated adenocarcinoma during surgery at the Zhongshan Hospital of Fudan University, which was approved by the Clinical Research Ethics Committee of Zhongshan Hospital of Fudan University. The patients were diagnosed and the tissue specimens were histologically checked using microscopy by pathologic experts, and the clinic and pathological data were accessible in Ref. [47]. The fibroblasts had been characterized using *in vitro* coculture and *in vivo* xenograft mouse model [7]. These cells were maintained in DMEM/F12. Human colon fibroblast cell line CCD-18Co was purchased from the American Type Culture Collection (ATCC). Human colon cancer cell lines LoVo and SW620 were bought from the Cell Bank of Shanghai Institutes for Biological Sciences, China. LoVo cells were cultured in RPMI 1640 supplied with 10% FBS. HT-29 cells were incubated with McCoy's 5A medium (Gibco, Shanghai, China)/10% FBS. SW620 cells were cultured in DMEM supplemented with 10% FBS. CCD-18Co and cultured in MEM/EBSS (Hyclone, Shanghai, China) supplemented with 10% FBS. Penicillin and streptomycin (Hyclone, Shanghai, China) (1%) were added to all culture media. Cells were all cultured in a humidified incubator at 37°C, 5% CO_2_.

### Antibodies

Antibodies against human alpha-smooth muscle actin (α-SMA) and vimentin (VIM) were purchased from PTGLAB, Wuhan, China. Antibody of human platelet-derived growth factor receptor alpha (PDGFRα) was bought from Merck Millipore, Shanghai, China. Antibodies for collagen alpha-1(IV) chain (COL4A1), filamin C (FLNC), COL6A3 and spondin-2 (SPON2) were bought from Sigma-Aldrich, Shanghai, China. Antibody of glyceraldehyde-3-phosphate dehydrogenase (GAPDH) was purchased from Kangwei Century Co. LTD, Beijing, China.

### Conditioned medium (CM) collection

Fibroblasts or colon cancer cells were seeded in 100 mm plates at 3–4 × 10^6^/plate and incubated until reaching 70–80% confluence. The cells were rinsed four times with serum-free and phenol red-free DMEM/F12 (Hyclone, Shanghai, China). The cells were cultured in this medium for 48 h. The CM was harvested and cleared by low speed centrifugation to remove cellular debris. The CM was further filtered using a 0.45 μm filter (Millipore, Shanghai, China). The CM was concentrated by ultrafiltration with Amicon^®^ Ultra 4 mL, 3 kDa Filters (Millipore, Shanghai, China).

### Protein quantitation and Western blot

Protein quantification was performed using a bicinchoninic acid assay (BCA) protein quantitation kit (Beyotime Inc., Haimen, China). Proteins were separated by 10% SDS-PAGE and transferred onto Immobilon-P Transfer Membrane (Merck Millipore, Shanghai, China). Immunoblots were performed using a standard protocol. Freshly prepared SuperSignal West Femto Maximum Sensitivity Substrate (Thermo Scientific Pierce, Shanghai, China) was used for chemiluminescent detection with a LAS-3000 imager (Fujifilm, Shanghai, China).

### Isobaric tags for relative and absolute quantitation (iTRAQ) labeling

ITRAQ labeling was performed using the 8-plex iTRAQ reagents (AB SCIEX, Shanghai, China) according to the manufacturer's instruction with minor modifications. Briefly, 100 μg secreted proteins in 20 μL from the eight cell lines were treated with Denaturant reagent (1 μL), followed by treating with reducing reagent (2 μL) for 1 h at 60°C. Free cysteine residues were blocked with Blocking Reagent. Trypsin was added to the mixture in a 40:1 (w/w) ratio and proteins were digested for 13 h at 37°C with gently agitation, followed by an additional digestion for 3 h by adding trypsin in a 100:1 ratio. The pH of the mixture was adjusted to 7 ∼ 9 with Dissolution buffer and low concentration of NH_4_HCO_3_. The digested peptides of each sample were labeled with 8-plex iTRAQ reagents for 2 h at room temperature. The labeled peptides were combined, lyophilized and stored at −20°C before further processing. The labeling peptides were fractionated into 25 fractions using strong cation chromatography (SCX) with a Polysulfoethyl column (2.1 mm (diameter) × 100 mm (length), packed with 200 Å and 5 μm materials) (The Nest Group, Inc., Shanghai, China). The fractions with high salt were desalted using a Sep-Pak Vac C18 column (Waters). Finally, the samples were lyophilized.

### Liquid chromatograph-mass spectrometry (LC-MS)

The iTRAQ samples were analyzed using LC-MS for triple times. Each fraction from the first dimensional separation was reconstructed with the loading buffer and loaded onto a ZORBAX 300SB-C18 column (packed with 300 Å, 5 μm C18 materials, 0.5 (diameter) × 23 mm (length)) (Waters, Shanghai, China) for concentration and desalting. Mobile phase A containing 5% ACN and 0.1% formic acid (FA) and mobile phase B containing 95% ACN/0.1% FA were delivered by a Nano Aquity UPLC system (Waters, Shanghai, China). The peptides were then loaded onto a PepMap reverse phase analytical column (packed with 100 Å and 3 μm C18 materials, 75 μm (diameter) × 150 mm (length)) (Dionex, Shanghai, China). The peptides were eluted into a Qstar Elite mass spectrometer (Applied Biosystems, Shanghai, China) with a linear 90-min gradient from 5 to 45% buffer B at a flow rate of 400 nL/min. A 10-mm id PicoTip nanospray emitter (New Objective, Shanghai, China) was connected to the end of the analytical column and used for ionization and steady spray. An acquisition duty cycle starts with one survey scan at a m/z range from 400 to 1800 Da in 0.5 sec, followed with 6 tandem mass spectrometry scan at a m/z range from 100 to 1800 Da in 0.5 or 1 sec with enhancement for all MS range. The top three ion exceeded 50 counts with charge 2+, 3+ or 4+ were chosen for fragmentation with automatic collision energy. An exclusion window avoiding re-acquisition was set as 180 sec. The LC-MS was controlled using software Analyst QS 2.0 (Applied Biosystems, Shanghai, China) and operated in positive mode.

### Database (DB) searching

Raw data processing and DB searching was performed using ProteinPilot v2.5 (Applied Biosystems, revision number: 1656). The searching DB contained all human proteins (20,214 entries) extracted from UniProtKB/Swiss-Prot Release 2014_06 with “Homo sapiens” as a constraint using an in-house PERL script. The DB searching was performed using Paragon method (algorithm version 4.5.0.0, 1654). In this searching method, the digestion enzyme trypsin and the thorough identification search effort were applied. The fixed cysteine alkylation modification is defined as methyl methanethiosulfate (MMTS). Bias correction and background correction were applied. The sample type was defined as iTRAQ 8-plex (Peptide Labeled). The denominator was iTRAQ 113. The Biological modifications were chosen for the ID focus. To meet the requirement of a further false discovery rate (FDR) analysis, the detected protein threshold, also termed as the Unused ProScore (Conf), was set at 0.05 which corresponds to a confidence of 10%. In addition, the competitor error margin, a ProtScore, was defined as 2.00. A further independent FDR analysis was performed by the PSPEP algorithm which was integrated in the ProteinPilot software. Other settings, like the missed cleavage site, mass tolerances of precursor or fragment ions and variable modifications were allowed as default as built-in functions of the ProteinPilot software. The DB searches for the three iTRAQ LC-MS analyses were performed independently and the raw protein identification results including all decoy matches were combined according to the protein accession numbers.

The primary results were filtered based on protein level FDR and peptide probability, which were determined by ProteinPilot and PSPEP algorithm. The 1% FDR at protein level were corresponding to unused ProScores 2, 1.62 and 2.01 for the three iTRAQ analyses, respectively (Supplementary Table 3). An accepted protein must has an unused score above 1% FDR and contains at least 2 unique peptides of 99% probability in at least one of the three LC-MS analyses. This stringent setting filtered all decoy matches, achieving an overall FDR of 0%. The protein and spectrum identification results were deposited in Supplementary Table 1 and 2, respectively.

The raw quantitative values were iTRAQ ratios expressed as 113:113 (equal to 1), 114:113, 115:113, 116:113 and 117:113 for the fibroblast cells and 118:113, 119:113 and 121:113 for the colon cancer cells. The iTRAQ ratios were averaged for the fibroblast group and cancer group, respectively. Next, a fold change (FC) was calculated based on the two average values. A *p* value was calculated with the Student's *t* test and the *p* value < 0.05 was considered as significant. The FC and *p* value were calculated for the three LC-MS analyses independently. A protein was considered as fibroblast- or epithelial cell-enriched with a FC ≥ 2/*p* < 0.05 in all three iTRAQ replicates.

### Subcellular localization analysis

The flat text file of identified proteins were retrieved from UniProt DB (http://www.uniprot.org) and GO information of subcellular localization of each protein was extracted from the flat text. GO annotation of subcellular localization was also analyzed using the DAVID Bioinformatics Resources (http://david.abcc.ncifcrf.gov/) [48]. Classical secreted protein was predicted using SignalP v4.1 (http://www.cbs.dtu.dk/services/SignalP/) and Phobius (http://phobius.sbc.su.se/). Non-classical secreted protein without a signal sequence were predicted using SecretomeP v2.0 (http://www.cbs.dtu.dk/services/SecretomeP/). Protein localization was also evaluated using exosome databases including ExoCarta DB v4.1 (http://www.exocarta.org) (7234 human protein identification entries) [19] and Vesiclipedia DB v3.1 (http://www.microvesicles.org/) (72141 human protein identification entries) [20]. All of these information were integrated and manually checked and a final annotation was made for each protein (Supplementary Table 1). First, proteins were identified as a secreted protein when annotated as “secreted”, “extracellular region/space/matrix” or predicted to be a classic or non-classic secreted protein. Next, proteins with annotations like “plasma membrane”, “cell junction” or “cell membrane” were classified as plasma membrane proteins. The remaining proteins were categorized as intracellular proteins.

### Other bioinformatics analyses

We analyzed the mRNA expression level of identified proteins in colon cancer and other types of cancers using Oncomine cancer gene expression microarray DB (https://www.oncomine.org/resource/login.html). The *p* value was set up at 0.05. The fold change and gene rank were defined as “all”, whereas the data type was restricted to mRNA. Hierarchical cluster analysis was performed using the average linkage method by Cluster v3.06 (http://bonsai.hgc.jp/∼mdehoon/software/cluster/software.htm#ctv) and the heat map was displayed using TreeView v1.1.6r3 (http://sourceforge.net/projects/jtreeview/files/jtreeview/). Area-proportional Venn diagram was generated with BioVenn (http://www.cmbi.ru.nl/cdd/biovenn/index.php). GO enrichment analysis was performed using the DAVID Bioinformatics Resources (http://david.abcc.ncifcrf.gov/) with enrichment threshold of 0.05 (Supplementary Table 4). Over-represented diseases and bio functions of identified colonic stroma-enriched or epithelial cell-enriched proteins were revealed using IPA (Ingenuity Systems, Mountain View, CA, USA) (http://www.ingenuity.com) with default parameters (Supplementary Table 5).

### IHC and staining intensity analysis

All IHC analyses were performed using a commercial TMA (catalog no. HCol-Ade180Sur-04, Shanghai Outdo Biotech, China), which includes 90 pairs of colorectal adenocarcinoma and normal counterpart tissues (Supplementary Table 6). The caners were classified as stage I (9 cases), II (47), III (31) and IV (2) according to the 7^th^ the American Joint Committee on Cancer/International Union against cancer classification. The operations were carried out within July 2006 to May 2007. The survival time is ranging from 3 to 85 months with a median of 51 months. There were 46 dead and 44 alive until the last follow-up time August 2013. Follow-up informations are missing for 7 cases. IHC analysis was performed with the COL6A3 antibody at a dilution of 1:100 according to a commercial protocol of Outdo Biotech. The staining substrate was 3,3′-diaminobenzidine (DAB) and the nuclei were counterstained with hematoxylin. The TMA was scanned using Scanscope XT (Aperio, Shanghai, China). Scoring of the COL6A3 expression was performed based on staining intensity scanned using software Image-Pro Plus 6.0. First, the sample spot images were exported using Aperio ImageScope v. 12. To analyze the COL6A3 expression in the stromal cells, epithelial cells were excluded by filling with white color using Image-Pro Plus. Similarly, the stromal regions were blocked if the epithelial areas were analyzed. The IOD of the dark brown color in each image using the Image-Pro Plus. The parameters for intensity calibration and image segmentation were optimized with one of the images and were applied for all images. Next, the intensity values (IOD/area) of different images from the same sample were averaged. The samples were ranked from the lowest to highest intensity values and a cutoff (0.043) to separate the negative and positive staining was determined manually by checking the whole staining of each sample. Samples without follow-up information or with no visible stromal or epithelial cells were excluded from analysis.

### Statistics

To analyze the relationship of *COL6A3* mRNA expression with CRC patients, the expression values of *COL6A3*, survival time and survival status in datasets containing survival information were retrieved from the Oncomine database. The samples were divided into higher-than-median and lower-than-median groups and the Kaplan-Meier survival curves were calculated using the log-rank test with GraphPad Prism 6.01. By the TMA-IHC assay, the survival analysis of CRC patients was performed based on COL6A3 positive or negative staining of the stromal or epithelial region of CRC tissues. The correlation between COL6A3 expression and the clinicopathological parameters were evaluated by Fisher's exact test or χ^2^ test using PASW Statistics 18. The significance between the normal and cancerous samples was calculated using two-tailed Student's *t* test. A *p* value < 0.05 was considered as significant.

### ELISA and ROC curve analysis

The plasma samples from CRC patients and healthy donors were collected in the First Affiliated Hospital of Soochow University, Jiangsu, China, with informed consent signed by patients. The available clinic information was listed in Supplementary Table 8. The analysis was permitted by the Clinical Research Ethics Committee of the First Affiliated Hospital of Soochow University. ELISA was performed using 5-fold diluted plasma samples with an ELISA kit purchase from Walan Biotechnology, Inc., Shanghai, China. COL6A3 concentration was determined using standard curve. ROC curve analysis was performed using PASW Statistics 18. The optimal concentration of COL6A3 for diagnosis purpose was determined based on the maximal sensitivity and specificity.

## SUPPLEMENTARY FIGURE AND TABLES













## Abbreviations

α-SMA: alpha-smooth muscle actin
ATCC: American Type Culture Collection
BCA: bicinchoninic acid assay
BMP7: bone morphogenetic protein 7
CAF: cancer-associated fibroblast
CM: conditioned media
COL12A1: collagen XII
COL4A1: collagen alpha-1(IV) chain
COL6A3: collagen alpha-3(VI) chain
CRC: Colorectal cancer
CTHRC1: collagen triple helix repeat-containing protein 1
DAB: 3,3′-diaminobenzidine
DB: database
DKK3: dickkopf
FDR: false discovery rate
FLNA: filamin A
FLNC: filamin C
FN3: fibronectin type 3 domain
FSTL1: follistatin-related protein 1
GAPDH: glyceraldehyde-3-phosphate dehydrogenase
GO: gene ontology
HDGF: hepatoma-derived growth factor
IHC: Immunohistochemistry
IOD: integrated optical density
IPA: Ingenuity Pathway Analysis
iTRAQ: isobaric tags for relative and absolute quantitation
KU: BPTI/Kunitz family of serine protease inhibitors
LC-MS: liquid chromatograph-mass spectrometry
MMP3: matrix metallopeptidase 3
MMTS: methyl methanethiosulfate
MSCs: mesenchymal stem cells
PDGFRα: platelet-derived growth factor receptor alpha
PSPEP: ProteomicS Performance Evaluation Pipeline Software
SCX: strong cation chromatography
SILAC: stable isotope labeling with amino acids in cell culture
SPON2: spondin-2
THBS2: thrombospondin 2
TMA: tissue microarray
VIM: vimentin
vWA: Von Willebrand factor type A
