# Supplementary material for: Stroma derived COL6A3 is a potential prognosis marker of colorectal carcinoma revealed by quantitative proteomics
Source: Oncotarget. 2015 Aug 12;6(30):29929–46. doi: 10.18632/oncotarget.4966 (PMC4745773; doi:10.18632/oncotarget.4966)
Supplement: Supplementary file 1 [file oncotarget-06-29929-s001.pdf]

SUPPLEMENTARY FIGURE AND TABLES

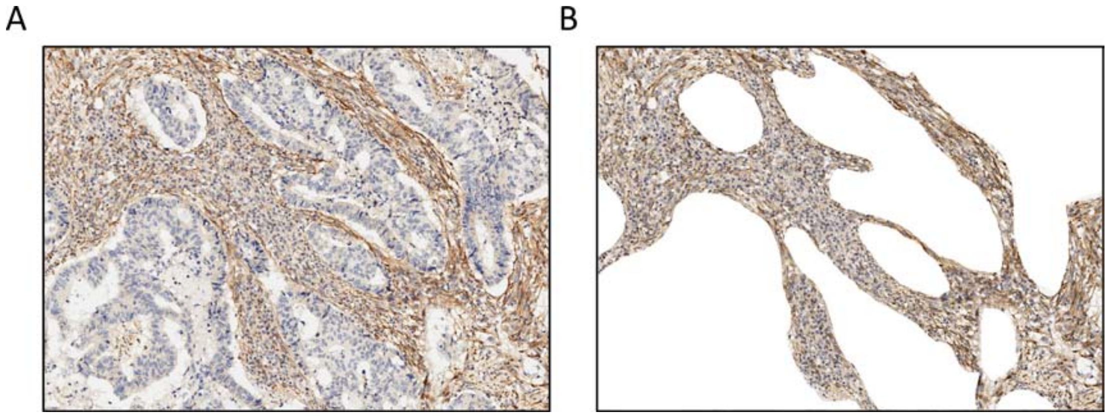

Supplementary Figure S1: The epithelial region was blocked before the integrated optical density (IOD) analysis of the stromal region. A. A local picture picked from one CRC sample showing the cancerous epithelial cells and the adjacent stromal cells. **B.** The epithelial regions were filled with white color and only the stromal region was subjected to IOD analysis by Image Pro Plus.

Supplementary Table S1: FDR analysis results performed by the built-in ProteomicS Performance Evaluation Pipeline Software (PSPEP) in ProteinPilot.

Supplementary Table S2: iTRAQ analysis of the secreted proteins from the colonic fibroblasts and colon cancer cell lines.

Supplementary Table S3: Gene Ontology enrichment analysis of identified fibroblast- or epithelial cancer cell-enriched proteins.

Supplementary Table S4: Ingenuity Pathway Analysis (IPA) of identified fibroblast- or epithelial cancer cell-enriched proteins.

Supplementary Table S5: The clinicopathological factors of the colorectal cancer patients in the tissue microarray.

Supplementary Table S6: Correlation between the mRNA expressions of *COL6A3* and clinicopathological variables in patients with colorectal cancer revealed by datamining of Oncomine gene array datasets

Oncomine dataset	Clinicopathological parameters	Total cases	mRNA expression		X^2	*p* value
			Lower-than-median	Higher-than-median		
**Bittner Colon**						
	**Dukes Stage**					
	A	49	32	17	13.596	0.001
	B	101	51	50		
	C-D	87	29	58		
	**Smoking Status**					
	Never Smoker	179	101	78	5.448	0.02
	Smoker	194	86	108		
	**Stage**					
	0-I	52	35	17	14.951	0.002
	II	103	49	54		
	III	85	30	55		
	IV	88	49	39		
	**T stage**					
	Tis-T1	15	12	3	9.311	0.025
	T2	55	31	24		
	T3	220	97	123		
	T4	28	15	13		
	**Tobacco Use**					
	Yes	194	86	108	5.448	0.02
	No	179	101	78		
**Smith Colorectal**						
	**Recurrence Status**					
	Recurrence	36	11	25	7.502	0.006
	No Recurrence	109	62	47		

The anlaysis was performed using datasets from the Oncomine cancer gene expression microarray DB (https://www.oncomine.org/resource/login.html).

Supplementary Table S7: Correlation between stromal or epthelical expression of COL6A3 and clinicopathological variables of 90 colorectal cancer cases used in TMA-IHC analysis

	Stroma expression					Epithelia expression				
Clinicopathological parameters	Total cases	COL6A3 (−)	COL6A3 (+)	X^2 or Fisher's exact test	p^a	Total cases	COL6A3 (−)	COL6A3 (+)	X^2 or Fisher's exact test	p^a
**Gender**										
Male	44	14	30		**0.268**	44	16	28		**0.8234**
Female	42	19	23			42	14	28		
**Age**										
≤ 70	45	16	29		**0.506**	45	15	30		**0.8221**
> 70	39	17	22			39	14	25		
**Grade**										
I	5	2	3	1.951, 2	0.377	5	0	5	3.065, 2	0.216
II	47	21	26			47	17	30		
III	34	10	24			35	14	21		
**Positive Lymph Nodes**										
0	48	18	30		**1**	48	18	30		**0.81**
1–12	31	12	19			31	10	21		
**T stage**										
T1	3	2	1	1.379, 3	0.7105	3	1	2	1.002, 3	0.8007
T2	6	2	4			6	1	5		
T3	65	24	41			65	23	42		
T4	10	3	7			10	4	6		
**N stage**										
N0	54	20	34	1.903, 2	0.3862	54	19	35	0.01179, 2	0.9941
N1	23	11	12			23	8	15		
N2	9	2	7			9	3	6		
**M stage**										
M0	83	31	52		**1**	83	30	53		**0.5378**
M1	2	1	1			2	0	2		
**Stage**										
1	7	4	3	2.151, 3	0.5417	7	2	5	0.1312, 2	0.9365
2	45	14	31			45	16	29		
3	30	12	18			32	11	21		
4	2	1	1			2	0	2		

(*Continued*)

	Stroma expression					Epithelia expression				
Clinicopathological parameters	Total cases	COL6A3 (−)	COL6A3 (+)	X^2 or Fisher's exact test	p^a	Total cases	COL6A3 (−)	COL6A3 (+)	X^2 or Fisher's exact test	p^a
**Survival time (month)**										
≤40	30	7	23		**0.033**	30	10	20		**1**
>40	49	24	25			49	16	33		
**Survival status**										
Dead	42	12	30		**0.08**	42	15	27		**1**
Alive	44	21	23			44	15	29		

[a]The bold *p* values were calculated using Fisher's exact test.

Supplementary Table S8: Plasma samples used for ELISA analysis of COL6A3 concentration

Sample code	Sample types	Sex	Age	Clinical diagnosis	Pathologic diagnosis	Lymph node metastasis
C1	Colon cancer	Male	78	Colon cancer	Adenocarcinoma	Yes
C2	Colon cancer	Female	61	Colon cancer	Adenocarcinoma	Yes
C3	Colon cancer	Male	64	Colon cancer	Adenocarcinoma	Yes
C4	Colon cancer	Female	57	Colon cancer	Adenocarcinoma	No
C5	Colon cancer	Male	47	Colon cancer	Adenocarcinoma	Yes
C6	Colon cancer	Male	82	Colon cancer	Adenocarcinoma	Yes
C7	Colon cancer	Male	82	Colon cancer	Adenocarcinoma	Yes
C8	Colon cancer	Male	55	Colon cancer	Adenocarcinoma	No
C9	Colon cancer	Male	60	Colon cancer	Adenocarcinoma	No
C10	Colon cancer	Female	55	Colon cancer	Adenocarcinoma	No
C11	Colon cancer	Male	71	Colon cancer	Adenocarcinoma	No
C12	Colon cancer	Female	40	Colon cancer	Adenocarcinoma	Yes
C13	Colon cancer	Female	63	Colon cancer	Adenocarcinoma	Yes
C14	Colon cancer	Male	66	Colon cancer	Adenocarcinoma	No
C15	Colon cancer	Female	69	Colon cancer	Adenocarcinoma	No
C16	Colon cancer	Female	73	Colon cancer	Adenocarcinoma	No
C17	Colon cancer	Female	77	Colon cancer	Adenocarcinoma	Yes
C18	Colon cancer	Male	73	Colon cancer	Adenocarcinoma	No
C19	Colon cancer	Female	70	Colon cancer	Adenocarcinoma	Yes
C20	Colon cancer	Male	65	Colon cancer	Adenocarcinoma	No
C21	Colon cancer	Female	76	Colon cancer	Adenocarcinoma	n/a
C22	Colon cancer	Male	71	Colon cancer	Adenocarcinoma	No
C23	Colon cancer	Male	72	Colon cancer	Adenocarcinoma	No
C24	Colon cancer	Female	81	Colon cancer	Adenocarcinoma	No
C25	Colon cancer	Male	52	Colon cancer	Adenocarcinoma	Yes
C26	Colon cancer	Male	59	Colon cancer	Adenocarcinoma	No
C27	Colon cancer	Male	59	Colon cancer	Adenocarcinoma	Yes
C28	Colon cancer	Male	57	Colon cancer	Adenocarcinoma	No
C29	Colon cancer	Male	43	Colon cancer	Adenocarcinoma	No
C30	Colon cancer	Male	61	Colon cancer	Adenocarcinoma	n/a
C31	Colon cancer	Male	66	Colon cancer	Adenocarcinoma	Yes
C32	Colon cancer	Female	42	Colon cancer	Adenocarcinoma	No
C33	Colon cancer	Male	57	Colon cancer	Adenocarcinoma	Yes
C34	Colon cancer	Male	67	Colon cancer	Adenocarcinoma	Yes

(*Continued*)

Sample code	Sample types	Sex	Age	Clinical diagnosis	Pathologic diagnosis	Lymph node metastasis
C35	Colon cancer	Male	80	Colon cancer	Adenocarcinoma	No
C36	Colon cancer	Male	85	Colon cancer	Adenocarcinoma	No
C37	Colon cancer	Male	70	Colon cancer	Adenocarcinoma	No
C38	Colon cancer	Male	66	Colon cancer	Adenocarcinoma	No
C39	Colon cancer	Female	70	Colon cancer	Adenocarcinoma	Yes
C40	Colon cancer	Male	60	Colon cancer	Adenocarcinoma	No
C41	Colon cancer	Male	64	Colon cancer	Adenocarcinoma	No
C42	Colon cancer	Female	61	Colon cancer	Adenocarcinoma	
N26	Normal	Male	71	Physical examination	–	–
N27	Normal	Male	45	Physical examination	–	–
N28	Normal	Male	47	Physical examination	–	–
N39	Normal	Male	41	Physical examination	–	–
N40	Normal	Female	62	Physical examination	–	–
N41	Normal	Female	65	Physical examination	–	–
N42	Normal	Female	47	Physical examination	–	–
N43	Normal	Female	49	Physical examination	–	–
N44	Normal	Male	27	Physical examination	–	–
N45	Normal	Female	65	Physical examination	–	–
N46	Normal	Female	51	Physical examination	–	–
N47	Normal	Female	40	Physical examination	–	–
N48	Normal	Male	41	Physical examination	–	–
N1	Normal	Female	36	Physical examination	–	–
N2	Normal	Male	52	Physical examination	–	–
N3	Normal	Male	41	Physical examination	–	–
N4	Normal	Female	20	Physical examination	–	–
N5	Normal	Female	52	Physical examination	–	–
N6	Normal	Male	37	Physical examination	–	–
N7	Normal	Female	23	Physical examination	–	–
N8	Normal	Female	55	Physical examination	–	–
N9	Normal	Female	26	Physical examination	–	–
N10	Normal	Male	25	Physical examination	–	–
N11	Normal	Male	60	Physical examination	–	–
N12	Normal	Female	27	Physical examination	–	–
N13	Normal	Male	21	Physical examination	–	–

(*Continued*)

Sample code	Sample types	Sex	Age	Clinical diagnosis	Pathologic diagnosis	Lymph node metastasis
N14	Normal	Female	24	Physical examination	–	–
N15	Normal	Male	22	Physical examination	–	–
N16	Normal	Male	28	Physical examination	–	–
N17	Normal	Female	47	Physical examination	–	–
N18	Normal	Male	20	Physical examination	–	–
N19	Normal	Female	51	Physical examination	–	–
N20	Normal	Female	50	Physical examination	–	–
N21	Normal	Female	65	Physical examination	–	–
N22	Normal	Female	20	Physical examination	–	–
N23	Normal	Female	70	Physical examination	–	–
N24	Normal	Male	66	Physical examination	–	–
N25	Normal	Male	26	Physical examination	–	–
N26	Normal	Female	19	Physical examination	–	–
N27	Normal	Male	19	Physical examination	–	–
N28	Normal	Female	38	Physical examination	–	–
N29	Normal	Female	n/a	Physical examination	–	–
N30	Normal	Female	20	Physical examination	–	–
N31	Normal	Female	22	Physical examination	–	–
N32	Normal	Male	42	Physical examination	–	–
N33	Normal	Female	39	Physical examination	–	–
N34	Normal	Male	25	Physical examination	–	–
N35	Normal	Male	66	Physical examination	–	–
